# Identification of robust and disease-specific stromal alterations in spondyloarthritis synovitis

**DOI:** 10.1186/1479-5876-9-S2-O1

**Published:** 2011-11-23

**Authors:** Nataliya Yeremenko, Troy Noordenbos, Gemma Rigter, Juan Cañete, Paul-Peter Tak, Dominique Baeten

**Affiliations:** 1 Academic Medical Center of Amsterdam, Amsterdam, The Netherlands; 2 Hospital Clinic, Barcelona, Spain

## Background

The cellular and molecular pathways driving synovial inflammation and stromal remodeling in spondyloarthritis (SpA) remain largely unknown. As SpA and rheumatoid arthritis (RA) show clearly distinct patterns of structural remodelling, we conducted this study to identify pathways specific for SpA synovitis by an unbiased microarray screening approach of the inflamed synovial tissue in both conditions.

## Materials and methods

Synovial tissue samples were obtained by arthroscopy from untreated individuals with SpA, RA and gout. RNA was extracted and gene expression profiling was performed. Top differentially expressed genes were validated on three independent cohorts of SpA versus control samples by qPCR and immunohistochemistry. qPCR was also performed on paired SpA synovial biopsies before and after TNF blockade.

## Results

The microarray experiments identified a signature set of 359 genes that discriminated with high certainty between SpA and RA patients. Both, technical validation by qPCR on the same samples, and biological validation in an independent cohort of early, untreated SpA and RA yielded a strong correlation between the microarray and qPCR data. The gene signature was also consistent as pathway analysis revealed that top-ranking upregulated transcripts in SpA were related to myocyte/myofibroblast biology. Several of these genes, including alpha smooth muscle actin (αSMA), showed up to 100-fold upregulation in SpA versus RA. Analysis of gout versus SpA samples confirmed that these genes were specifically upregulated in SpA synovitis rather than downregulated in RA. Immunohistochemistry for αSMA identified increased expression in SpA versus RA in the intimal lining and synovial sublining layers. Double immunofluorescence and FACS analyses revealed a colocalization of αSMA and fibroblasts marker CD90. Finally, paired analysis of SpA samples before and after TNF blockade showed that the expression of these genes was not altered.

## Conclusions

This study identified a robust and disease-specific increase in myofibroblasts in SpA synovitis. The reason for this increase and the potential role of these cells in inflammation and, more importantly, structural remodelling in SpA are currently under investigation.

